# ^18^F-FDG PET/CT and Urothelial Carcinoma: Impact on Management and Prognosis—A Multicenter Retrospective Study

**DOI:** 10.3390/cancers11050700

**Published:** 2019-05-20

**Authors:** Fabio Zattoni, Elena Incerti, Fabrizio Dal Moro, Marco Moschini, Paolo Castellucci, Stefano Panareo, Maria Picchio, Federico Fallanca, Alberto Briganti, Andrea Gallina, Stefano Fanti, Riccardo Schiavina, Eugenio Brunocilla, Ilaria Rambaldi, Val Lowe, R. Jeffrey Karnes, Laura Evangelista

**Affiliations:** 1 Department of Surgery, Oncology and Gastroenterology, University of Padua, 35128 Padua, Italy; fabrizio.dalmoro@gmail.com; 2 Urology Unit, Academical Medical Centre Hospital, 33100 Udine, Italy; 3 Nuclear Medicine Department, IRCCS San Raffaele Scientific Institute, 20132 Milan, Italy; incerti.elena@hsr.it (E.I.); picchio.maria@hsr.it (M.P.); fallanca.federico@hsr.it (F.F.); 4 Department of Urology, IRCCS San Raffaele Scientific Institute, 20132 Milan, Italy; marco.moschini87@gmail.com (M.M.); briganti.alberto@hsr.it (A.B.); gallina.andrea@hsr.it (A.G.); 5 Department of Nuclear Medicine, Sant’Orsola-Malpighi Hospital, 40138 Bologna, Italy; paolo.castellucci@aosp.bo.it (P.C.); stefano.fanti@aosp.bo.it (S.F.); 6 Nuclear Medicine Unit, Diagnostic Imaging e Laboratory Medicine Department, University Hospital of Ferrara, 44121 Ferrara, Italy; s.panareo@ospfe.it (S.P.); i.rambaldi@ospfe.it (I.R.); 7 Vita-Salute San Raffaele University, 20132 Milan, Italy; 8 Department of Urology, Sant’Orsola-Malpighi Hospital, 40138 Bologna, Italy; rschiavina@yahoo.it (R.S.); eugenio.brunocilla@aosp.bo.it (E.B.); 9 Division of Nuclear Medicine, Mayo Clinic, Rochester, MN 55905, USA; vlowe@mayo.edu; 10 Department of Urology, Mayo Clinic, Rochester, MN 55905, USA; Karnes.R@mayo.edu; 11 Nuclear Medicine and Molecular Imaging Unit, Veneto Institute of Oncology IOV—IRCCS, 35128 Padua, Italy; laura.evangelista@iov.veneto.it

**Keywords:** PET/CT, urothelial carcinoma, bladder cancer, upper tract urothelial carcinoma, survival

## Abstract

*Objectives*: To evaluate the ability of ^18^F-labeled fluoro-2-deoxyglucose positron emission tomography/computed tomography (^18^F-FDG PET/CT) to predict survivorship of patients with bladder cancer (BC) and/or upper urinary tract carcinoma (UUTC). *Materials*: Data from patients who underwent FDG PET/CT for suspicion of recurrent urothelial carcinoma (UC) between 2007 and 2015 were retrospectively collected in a multicenter study. Disease management after the introduction of FDG PET/CT in the diagnostic algorithm was assessed in all patients. Kaplan-Meier and log-rank analysis were computed for survival assessment. A Cox regression analysis was used to identify predictors of recurrence and death, for BC, UUTC, and concomitant BC and UUTC. *Results*: Data from 286 patients were collected. Of these, 212 had a history of BC, 38 of UUTC and 36 of concomitant BC and UUTC. Patient management was changed in 114/286 (40%) UC patients with the inclusion of FDG PET/CT, particularly in those with BC, reaching 74% (*n* = 90/122). After a mean follow-up period of 21 months (Interquartile range: 4–28 mo.), 136 patients (47.4%) had recurrence/progression of disease. Moreover, 131 subjects (45.6%) died. At Kaplan-Meier analyses, patients with BC and positive PET/CT had a worse overall survival than those with a negative scan (log-rank < 0.001). Furthermore, a negative PET/CT scan was associated with a lower recurrence rate than a positive examination, independently from the primary tumor site. At multivariate analysis, in patients with BC and UUTC, a positive FDG PET/CT resulted an independent predictor of disease-free and overall survival (*p* < 0,01). *Conclusions*: FDG PET/CT has the potential to change patient management, particularly for patients with BC. Furthermore, it can be considered a valid survival prediction tool after primary treatment in patients with recurrent UC. However, a firm recommendation cannot be made yet. Further prospective studies are necessary to confirm our findings.

## 1. Introduction

Bladder carcinoma (BC) is the fourth most common tumor in men, with an incidence of 146,650 new cases in the US every year. It accounts for 90–95% of urothelial carcinomas (UC) and it is the most common malignancy of the urinary tract [1]. Two percent of all cancer deaths in the United States are due to BC [1]. For 35% of patients with invasive BC diagnosed at a localized stage, the 5-year survival rate is 70%. However, survivorship drops from 81% to 47% for BC with non-muscle-invasive and with muscle-invasive disease [2]. In contrast, upper urinary tract carcinoma (UUTC) is uncommon and accounts for only 5–10% of UCs [3]. Upper tract urothelial carcinomas that invade the muscle wall usually have a poor prognosis. The 5-year specific survival rate is <50% for patients with pT2/pT3 tumors and <10% for those with pT4 [4,5].

Half of the patients with muscle-invasive UC relapse after surgery, depending on the pathological stage of the primary tumor and nodal status. Local recurrence accounts for 30% of relapses, whereas distant metastases are more common. Ten to fifteen percent of patients are already metastatic at diagnosis [6]. Before the development of effective chemotherapy, patients with metastatic urothelial cancer rarely had a median survival that exceeded 3–6 months [7].

Unfortunately, more than 50% of metastases are diagnosed after the appearance of symptoms despite the periodic monitoring with advanced imaging, including Computed Tomography (CT) and Magnetic Resonance Imaging (MRI) [8,9]. This is because recurrence patterns of UC after primary treatment are poorly predictable [10,11]. Thus, a major concern of follow up strategies after primary treatment is whether imaging can lead to the diagnosis of disease recurrence and, if so, how this may affect long-term survival [12]. These uncertainties during follow up may be justified by several factors, including:

(1) Available salvage treatments may be ineffective. Adjuvant chemotherapy after RC for patients with pT3/4 and/or lymph node positive (N+) disease without clinically detectable metastases (M0) is under debate [13] and is still infrequently used [14]. There is limited evidence from adequately conducted and accrued randomized phase III trials in favor of the routine use of adjuvant chemotherapy. From the currently available evidence, it is still unclear whether immediate adjuvant chemotherapy or chemotherapy at the time of relapse is superior, or if the two approaches are equivalent with respect to the endpoint of overall survival. Cisplatin-based combination chemotherapy results in long-term disease free survival, even in metastatic disease, mainly in patients with lymph node metastases only and with a good performance status [15]. Radiation therapy or salvage surgery are currently not an option for treatment of recurrence, but only for palliation.
(2) Existing biomarkers and conventional imaging accuracy may be insufficient in the assessment of lymph node involvement and distant metastasis. Indeed, guidelines do not recommend the use of biomarkers in daily clinical practice since they have no impact on predicting outcome, making treatment decisions, or monitoring therapy.

As a consequence, it is unclear what the best follow-up schedule on UC is and what the best imaging modalities are to diagnose disease recurrence and progression [12]. Preliminary studies on accuracy showed that positron emission tomography/ computed tomography (PET/CT) is a useful tool for restaging suspected UC relapse, especially in the assessment of lymphonodes (LN ) or distant metastases [16,17]. PET/CT accuracy of these retrospective studies are comparable with an overall good performance of PET/CT at patient-based analysis [17].

Interestingly, only a few studies have evaluated the role of ^18^F-labeled fluoro-2-deoxyglucose positron emission tomography/computed tomography (^18^F-FDG PET/CT) as a predictive tool for UC progression [18], while how PET/CT may change a patient’s surgical and medical treatment is still underreported. This has been shown in other urological cancers, such as prostate cancer [19]. PET/CT has a consolidated role in restaging after primary treatment of prostate cancer [20,21] and has also been introduced as a guide for salvage surgery (salvage lymph node dissection) [19]. Therefore, based on the above-mentioned limitations, the aim of this study was to evaluate the role of ^18^F-FDG PET/CT both in the management and in the survival prediction of patients with UC.

## 2. Materials

### 2.1. Study Approval and Patient Population

The study protocol was approved at IOV Institute on April 2016 (approval nr. 005275). Major US and European urological centers with experience in BC and PET/CT were asked to participate in the study. The centers that accepted and had available cases were provided with a dedicated Microsoft Excel file created for the purpose of the study. A computerized databank was generated to transfer data of anonymized patients. A retrospective database was then built with all patients who fit the inclusion and exclusion criteria of the study. After combining the data sets, reports were generated for each variable to identify data inconsistencies and other data integrity problems. Through regular communication with all sites, resolution of all identified anomalies was achieved before analysis. The database was then frozen, and the final data set was produced for the current analysis.

### 2.2. Patient Population

FDG PET/CT scans of 286 patients with suspected recurrent UC, collected by San Raffaele Hospital in Milan (Italy), Mayo Clinic in Rochester (MN, USA), Veneto Institute of Oncology IOV–IRCCS in Padua (Italy), Sant’Orsola Malpighi Hospital in Bologna (Italy), and Hospital of Ferrara (Italy), were retrospectively reviewed from 2005 to 2015. The same population was already included in another study assessing FDG PET/CT accuracy compared to conventional imaging [17]; however, a few patients were excluded because they did not met the inclusion criteria.

Inclusion criteria for the study were: (1) a known history of BC and/or in UUTC; (2) at least one FDG PET/CT for disease restaging (suspicion of recurrent disease or doubtful conventional imaging findings) after primary treatment (independently from the type of therapy); (3) the availability of images from conventional imaging modalities (abdominal ceCT or MRI, or total body ceCT, and chest X-ray), and (4) the availability of information on mid-long term follow-up after PET/CT imaging. Exclusion criteria were other abdominal tumors and chemotherapy administration concomitant to imaging and non-UC variants. For each patient, the following variables were collected: demographic data (age, sex, body mass index-BMI), clinical data (history of bladder cancer, last clinical stage, history of radical cystectomy, pTNM stage, history of UUTC, UUTC location in the upper tract: pelvis, ureter, multifocal), UUTC treatment (nephroureterectomy, endourology, other conservative surgery), and use and type of neoadjuvant treatments.

### 2.3. PET/CT Equipment and Image Acquisition Protocol

A standard comparable protocol was used in all centers for PET/CT image acquisition. All patients fasted for at least 6 h prior to imaging, and blood glucose levels were <180 mg/dL at the time of tracer injection. To minimize FDG uptake in skeletal muscles, all patients were instructed to avoid talking, chewing or any other muscular activity before undergoing PET/CT scan. PET/CT studies were acquired with integrated PET/CT systems, according to different study protocols in accordance with each participating Institution. PET data of the whole-body tracer distribution were then acquired (3 min per bed) in 3-D mode starting 60 min after i.v. administration of FDG. Attenuation correction was performed using CT images. CT and PET images were matched and fused into transaxial, coronal, and sagittal images. A positive PET scan was defined in the presence of pathological FDG uptake outside the areas of physiological biodistribution, later confirmed by co-registered CT abnormalities. The definition of true positive (TP), true negative (TN), false positive, and false negative are described elsewhere [15].

### 2.4. Change in Management

A change in patient management was defined as a modification of treatment or follow-up modalities after PET/CT scan compared to conventional imaging. For example, a shift from local therapies (surgery or radiotherapy) to systemic therapy or from systemic therapy to an observational period/palliation after PET/CT scan were both considered a modification of patient strategy. The number of patients whose management was changed after PET/CT scan were assessed separately for BC and UUTC. Change in management was based on the data available at the time of PET/CT, including the original interpretation, and was determined by the authors with a review of electronic medical records.

### 2.5. Follow Up

Patients were generally observed according to standard protocols at each institution: every three to six months for the first year after surgery, and annually thereafter. Follow-up consisted of a history, physical examination, routine blood work and serum chemistry studies, chest and abdominal conventional imaging. The follow-up data of selected patients were obtained from clinical charts. To determine follow-up time, the date of the last examination or consultation was used. Progression free survival (PFS) was defined as the length of time between imaging and disease progression. Cancer Specific Survival (CSS) was defined as the time from PET/CT to cancer specific death. Finally, Overall Survival (OS) was defined as the length of time between PET/CT and all-cause mortality. Death was confirmed by retrieval of death certificate.

### 2.6. Statistical Analysis

Categorical variables were reported as frequency, while continuous variables were reported as mean and standard deviation (SD) for variables with a normal distribution, and as median and interquartile range (IQR) for variables with a non-normal distribution. The Student’s T-test and the Mann–Whitney U-test were used to compare the locations of continuous variables, as appropriate. The Pearson chi-test was used to compare categorical variables. The survival intervals were defined as the time elapsed from surgery to the last clinical evaluation, the occurrence of UC recurrence, or patient death. PFS, CSS and OS were evaluated using Kaplan-Meier analysis. The log-rank test was used for comparison of the survival curves. PFS, CCS, and OS were compared for the entire population and then separately according to the site of the primary tumor and for the administration of adjuvant treatments.

Univariate and multivariate COX regression analyses were used to assess predictors of recurrence and survival. Two models were performed: in the first, data entry into the multivariable analysis was performed with a stepwise method when no variables were eligible to enter the model; only variables set at a *p* value less than 0.05 in the univariable analysis were included. As a second model, we included the most common clinical pathological variables such as age, sex (Male vs. Female), pT stage (pT ≤ 2 vs. pT3-4) pN stage (positive vs. negative), and PET/CT findings (positive vs. negative). A measure of goodness-of-fit was performed with a ROC analysis to evaluate the fit of all Cox regressions performed.

For some analyses, BC and UTCC were grouped together because they originate from transitional cells (which cover the entire urinary system) and they have the same risk factors, and finally, from epidemiological point of view, because UTUC often presents after BC. Analyses were performed using SPSS version 20 (IBM, Armonk, NY, USA).

## 3. Results

### 3.1. Clinical Characteristics

Of the 286 patients, 222 (77.6%) were male and 64 (22.4%) female. Mean age was 69 years (± SD: 10 years). The majority had BC only (*n* = 212; 86.8%), 38 (13.3%) had UUTC only and 36 (12.6%) had both BC and UUTC. Table 1 shows the characteristics of the patient population. Median follow up after primary tumor treatment and after PET/CT was 13 (5–28) and seven months (2–23), respectively.

### 3.2. Patient Management

**Figure 1:**
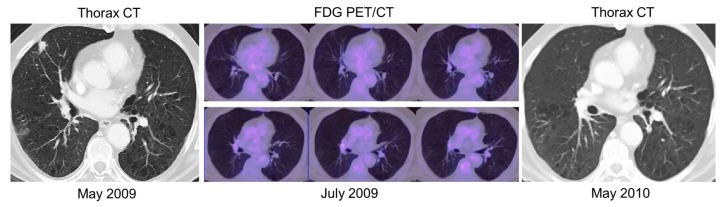
A 74 year-old man with a history of bladder cancer underwent thorax and abdominal contrast-enhanced CT (ceCT) scan, which showed suspected lung recurrence. Later, positron emission tomography/computed tomography (PET/CT) was negative for any recurrence, particularly in the lung. A further thorax-abdominal ceCT scan, performed after 1 year later, revealed the disappearance of the lung nodule. Therefore, PET/CT was able to change therapeutic management, from a potential curative intent to an observational strategy.

**Figure 2:**
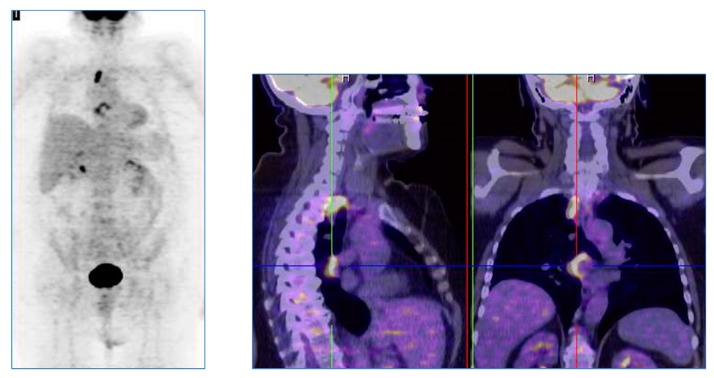
A 59 year-old woman with bladder cancer underwent abdominal contrast-enhanced CT (ceCT) for restaging of disease. CT images were considered completely negative. A positron emission tomography/computed tomography (PET/CT) performed after 3 months, for the persistence of suspicious symptomatology, showed a significant fluoro deoxyglucose uptake in the abdominal lymph nodes and in the liver, compatible with the presence of disease recurrence. The patient was later treated with systemic chemotherapy.

PET/CT was positive in 224 patients (175 with BC only and 49 with UUTC only or UUTC plus BC). PET/CT findings were compatible with local, lymph node, skeletal, lung and liver recurrence in 58 (25.9%), 125 (55.8%), 57 (25.4%), 53 (23.7%) and 26 (11.6%) cases, respectively. Patient management was changed in 114/286 (40%) cases by the inclusion of FDG PET/CT in the restage setting done with conventional imaging. In particular, a change in management was made in 90/122 pts (73.7%) with BC, 10/28 (35.7%) for UUTC and 14/22 (63.6%) for those with BC and UUTC. Of these patients, 33 (29%) received local therapies (surgery or radiotherapy), 43 (38%) chemotherapy, 33 (29%) a combination of local and systemic therapy and finally, five (4%) patients were observed without any therapy. Figure 1 and Figure 2 report two illustrative cases.

### 3.3. Follow Up

After a mean follow-up period of 21 months (IQR: 4–28 months) from primary treatment, 136 patients (47.4%) had recurrence/progression of disease and of these, 87 (63.9%) died. In total, 131 (45.8%) patients died, of whom 104 (79.4%) had a cancer-related death. A comparison of oncological outcomes among FDG PET/CT findings are reported in Table 2.

**Figure 3:**
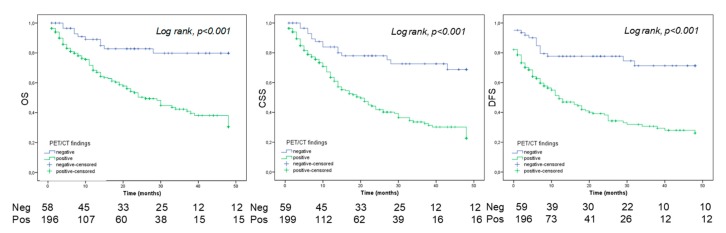
Overall survival (OS), cancer specific survival (CSS) and disease-free survival (DFS) for all patients (n = 286).

**Figure 4:**
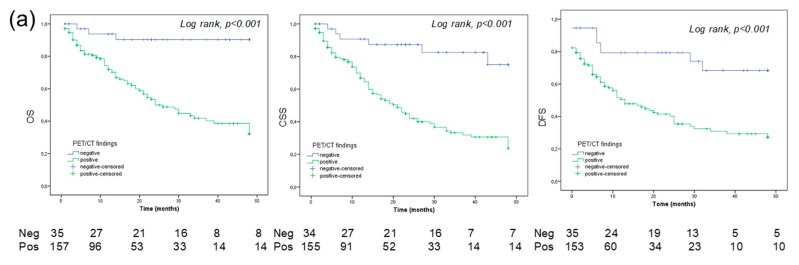

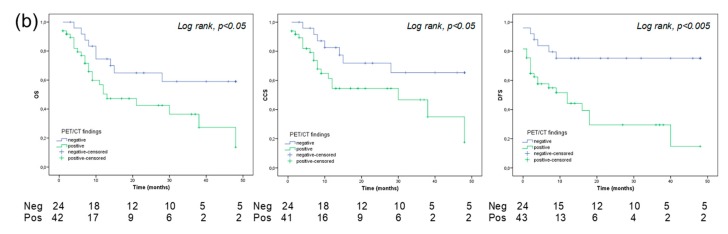
Overall survival (OS), cancer specific survival (CSS) and disease-free survival (DFS) for bladder cancer only (n = 212) (**a**) and for all patients with UTUC/UTUC and BC (n = 74) (**b**).

In all 286 patients, 5-year OS, CSS, and PFS estimates were 29%, 36%, and 28% in patients with a positive PET/CT, and 69%, 80%, and 71% in patients with a negative PET/CT, respectively (all log rank; *p* value *p* < 0.01; Figure 3). In patients with BC only, the 5-year OS, CSS and RFS estimates were 37%, 29%, and 29% in positive PET/CT group, and 90%, 75%, and 68% in the negative PET/CT group, respectively (log-rank, *p* value *p* < 0.01; Figure 4a).

In patients with UUTC and both UUTC and BC, the 5-year OS, CSS and RFS estimates were 14%, 18%, and 15% in positive PET/CT patients and 59%, 59%, and 75% in negative PET/CT subjects, respectively (Log rank, *p* value *p* < 0.05; Figure 4b).

The 5-year OS, CSS, and RFS were significantly worse in patients with a positive PET/CT than the counterpart regardless of the use of adjuvant/salvage treatments in follow up (Table 3).

Interestingly, PET/CT was able to stratify patients with a FP and FN findings at conventional imaging with survival analysis assessing OS and RFS (*p* < 0.01) (Figures S1 and S2).

### 3.4. PET/CT as a Predictive Tool

Table 4, Table 5 and Table 6 summarize univariable and multivariable Cox regression analyses assessing predictors of OS, CSS and PFS for BC, UUTC, and UUTC plus BC.

In a first model, using only the variables set at a *p* value < 0.05 in the univariable analysis (Table 4, Table 6 and Table 7), a positive FDG PET/CT, together with the presence of pT3–pT4, was an independent predictor of PFS, OS and CCS (*p* < 0.01) in patients with BC. In patients with either UUTC or concomitant BC and UUTC, a positive FDG PET/CT was a significant predictor of PFS, CCS, and OS only at univariate analysis.

In a second model, corrected for the most common clinical pathological variables (Table 5, Table 8 and Table 9), a positive FDG PET/CT in patients with BC was an independent predictor of PFS, OS, and CCS (*p* < 0.01). In the same model for patients with UUTC and those with concomitant BC and UUTC, a positive FDG PET/CT was an independent predictor of PFS (*p* < 0.01).

In the multivariate models, the inclusion of PET/CT increases the AUCs of the models (Figures S3–S7).

## 4. Discussion

PET/CT with FDG has been extensively used in solid cancers, but few papers are available on its use in urological cancer patients. FDG is highly expressed in aggressive tumors and its uptake is directly correlated with Glucose transporter 1 (GLUT1) expression. Some biological studies have demonstrated the relationship between glucose metabolism (i.e., GLUT 1 expression and others) and the proliferation of urothelial cancer [22]. Moreover, undifferentiated cancers, which are aggressive and associated with poor prognosis, are highly avid for glucose. These premises are essential for the employment of FDG PET/CT in this type of cancer. Our study finds two additional roles for PET/CT in patients with recurrent UC. First, PET/CT may predict the prognosis of recurrent UC patients. Second, PET/CT is able to change patient management in a high percentage of patients (40%) due to the detection of lymph node and distant metastases in recurrent disease.

The clinical impact of these two conclusions are reflected in the possibility to personalize treatment for patients with a positive vs. negative PET scan. In particular, patients with a negative scan may benefit from further follow up while those with a positive scan may benefit from further treatment to improve survival (both disease-free and overall survival). Furthermore, PET/CT may also play a role after systemic treatment for the evaluation of response to therapy, particularly in patients with doubtful findings on conventional imaging or those with a mismatch between clinical and radiological findings. We believe that the inclusion of FDG PET/CT in the restaging phase of patients with urothelial cancer will have a profound impact on the management of the disease. Furthermore, the sensitivity of FDG PET/CT was higher than 80% for patients affected by bladder cancer and UUTC, as demonstrated by our previous paper involving the same patient population [15]. Moreover, the diagnostic accuracy of FDG PET/CT resulted higher than CI in both subgroups of patients (91% vs. 81% in bladder cancer and 84% vs. 76% in UUTC, respectively for PET/CT and conventional Imaging.

To date, few studies with a limited number of enrolled patients have assessed the impact of FDG PET/CT on the therapeutic management in BC. Alongi et al. found, in a study involving 41 patients, a change in treatment after FDG PET/CT in about 40% of cases, the same as the present study [18]. In another study involving 57 patients, among which 72% underwent PET/CT during restaging, management was changed in 68% of cases [23], supporting a substantial impact of FDG PET/CT in the suspicion of recurrence.

Furthermore, a positive PET is a predictor of OS and PFS for both BC and UUTC. From our study, it emerged that a positive PET/CT is associated with a significantly higher rate of disease progression and a significantly reduced OS. On the other hand, a negative scan predicts a more favorable outcome. Interestingly, a 5-year OS was significantly favorable in patients with BC and a negative PET/CT compared to those with a positive scan, showing a gap of 53% (90% vs. 37%, respectively; see Figure 4a). Conversely, patients with UUTC have a worse 5-year OS survival, particularly when PET/CT was positive (gap: 45%). Moreover, in the predictive models, the inclusion of PET/CT increases the Area Under the ROC Curve (AUC), reaching a value of 0.83 in patients with UUTC. Furthermore, a positive PET/CT scan yielded the highest HR compared to other clinical pathological variables (see Table 4 and Table 5). These results are interesting because their implications may provide clinicians with what is still needed in the re-stage setting of UC: a decisional tool for active treatment (i.e., adjuvant chemotherapy) vs. follow up. Indeed, the identification of asymptomatic recurrence in patients who may benefit from salvage chemotherapy could significantly influence patient survival [24,25]. Nowadays, clinicians mainly suggest adjuvant therapy based on the well-known pathologic characteristics of the tumor, such as pT stage, grade, pathological lymph nodes, and comorbidities (i.e., Karnofsky performance status). Nomograms on CSS following radical cystectomy have been developed; however, their use is still not recommended until further data becomes available [26,27]. Interestingly, all these models try to predict survival in patients with disease recurrence following radical cystectomy without the use of imaging, which could have an essential role in the early evaluation of recurrence and in the prediction of disease outcome after primary treatment. It is now recognized that biomarker panels reflecting the biological heterogeneity of UCs can identify patients who may need aggressive therapies [28,29]. However, such panels have not been clinically implemented due to several shortcomings, including underpowered and clinically heterogeneous cohorts, short follow-up, use of non-disease-specific endpoints, and limited clinical validation. As a consequence, the development of clinically useful biomarkers to determine optimal treatment for patients remains somewhat elusive. Despite this, targeted therapy has demonstrated an OS benefit in the treatment of metastatic UC. As a new frontier, antibodies radiolabeled with positron-emitting radionuclides may allow Immuno-PET imaging with an in vivo quantification of the efficacy of targeted therapies. Thus, antibody targeted PET/CT may play a role in the selection of patient therapy, the prediction of response to systemic medications and an in vivo assessment of the efficacy of therapies (theragnostic) [30,31].

As any retrospective study, limitations are inherent. Different surgical treatments and follow up schedules may have been provided to patients at the different institutions during the study period. In the assessment of time dependent outcomes, we included patients treated with adjuvant therapies. Although this variable was adjusted in the multivariable analysis, patient imbalance and selection bias cannot be excluded. In some analyses, patients with BC and UUTUC were considered as one group. Although tumors differed, stage and grade were concordant in almost all cases. A selection bias could not be excluded considering the selected nature of the population and the use of the imaging test only in the event of suspicion of recurrence. Finally, images were not centrally reviewed, although all the radiologists at the time had at least 5 years of experience in genito-urinary imaging and FDG PET/CT.

## 5. Conclusions

This study assesses a promising role of FDG-PET/CT as a predictor of UC outcomes. Patient management was changed after FDG PET/CT in 40% of patients with UC. This may suggest a role for FDG-PET/CT in post-surgical therapeutic decision-making, although firm recommendations cannot yet be made. In particular, whether the present results are translated into better oncological outcomes still needs to be confirmed. It is crucial to prospectively investigate the clinical value of FDG-PET/CT in the assessment of UC recurrence.

## Figures and Tables

**Table 1:** Characteristics of patients with only bladder cancer (212/286), only UTUC (38/286) and concomitant bladder cancer and UTUC (36/286).

Variables	Only Bladder Cancer (212/286)	Only UTUC	Concomitant Bladder Cancer and UTUC (36/286)
		(38/286)	
Tumor grade			
Low Grade	15/212 (7.0)	1/38 (2.6)	2/36 (5.5)
High grade	197/212 (91.0)	27/38 (96.4)	30/36 (83.3)
N/A	4/212 (1.8)		4/36 (11.1)
pT of bladder cancer, n (%)			
pT ≤ 2	82/212 (40.0)		21/36 (58.3)
pT 3–4	126/212 (59.4)		11/36 (30.5)
NA	4/212 (1.8)		4/36 (11.1)
pN of bladder cancer, n (%)			
pNx	3/212 (1.4)		1/36 (2.8)
pN0	125/212 (59)		22/36 (61.1)
pN1	29/212 (13.7)		4/36 (11.1)
pN2	43/212 (20.3)		4/36 (11.1)
pN3	9/212 (4.2)		1/36 (2.8)
NA	3/212 (1.4)		4/36 (11.1)
pT of UUTC (*), n (%)			
pT ≤ 2		13/38 (34.2)	17/36 (47.2)
pT 3–4		16/38 (42.1)	12/36 (33.3)
NA		9/38 (23.7)	7/36 (19.4)
pN of UUTC (*), n (%)			
pNx		19/38 (50.0)	31/36(86.1)
pN0		14/38 (36.8)	4/36(11.1)
pN1		2/38 (5.2)	1/36 (2.8)
pN2		3/38 (7.9)	
Neoadjuvant treatments, n (%)	25/212 (11.8)	5/38 (13.1)	5/36 (13.9)
Adjuvant treatments, n (%)	93/212 (43.9)	8/38 (21.1)	10/36 (27.8)
Type of adjuvant treatments, n (%)			
No	119/212 (56.1)	30/38 (72.2)	26/36 (72.2)
Chemotherapy	75/212 (35.4)	7/38 (18.4)	7/36 (19.4)
Radiotherapy	17/212 (8)	0	2/36 (5.6)
Combination	1/212 (0.5)	1/38 (2.6)	1/36 (2.8)

UUTC: upper urinary tract carcinoma.

**Table 2:** Comparison of oncological outcomes between fluoro-2-deoxyglucose positron emission tomography/computed tomography (FDG PET/CT) findings.

	Negative PET/CT	Positive PET/CT	*p* Value *
Disease recurrence/progression, n (%)			
No	47 (75.8)	103 (46)	<0.001
Yes	15 (24.2)	121 (54)	
All-cause mortality, n (%)			
No	47 (75.8)	108 (48.2)	<0.001
Yes	15 (24.2)	116 (51.8)	
Cancer-related mortality, n (%)			
No	52 (83.9)	130 (58)	<0.001
Yes	10 (16.1)	94 (42)	

* Chi-square test.

**Table 3:** 5-year survival rates in all patient population stratified according to the use of adjuvant/salvage treatments during follow up, regardless the use of adjuvant/salvage treatments the 5-year OS, CSS and RFS were significantly worse in patients with a positive PET/CT.

		Negative PET/CT	Positive PET/CT	*p* Value *
No adjuvant/salvage treatments (n = 175 pts)	OS	0.669	0.258	<0.001
	CSS	0.805	0.318	<0.001
	RFS	0.631	0.210	<0.001
Adjuvant/salvage treatments (n = 111 pts)	OSS	0.743	0.300	<0.005
	CSS	0.805	0.398	<0.01
	RFS	0.614	0.224	<0.05

OS: overall survival; CSS: cancer specific survival; RFS: recurrence free survival, * Log-Rank test.

**Table 4:** Univariate and multivariate analysis in all patients (n = 286) assessing predictors of overall survival (OS), cancer specific survival (CCS) and progression free survival (PFS). Only variables set at a *p* value less than 0.05 in the univariable analysis were included in the model.

	OS						CCS						PFS					
	Univariate			Multivariate			Univariate			Multivariate			Univariate			Multivariate		
	HR	CI95%	p	HR	CI95%	p	HR	CI95%	p	HR	CI95%	p	HR	CI95%	p	HR	CI95%	p
Age	1.02	1.00–1.03	0.018	1.02	1.03–1.03	0.022	1.01	0.99–1.03	0.19	-	-	-	1.01	0.99–1.03	0.05	1.01	0.99–1.03	0.05
Gender	1.13	0.76–1.68	0.521	-	-	-	1.01	0.64–1.60	0.93	-	-	-	1.32	0.90–1.92	0.14	1.32	0.90–1.92	0.14
Neoadjuvant therapy	1.69	1.06–2.70	0.027	1.77	1.09–2.88	0.020	1.83	1.10–3.06	0.01	1.67	1.00–2.79	0.04	1.29	0.80–2.08	0.28	1.29	0.80–2.08	0.28
Adjuvant/salvage therapies	1.26	0.88–1.79	0.193	-	-	-	1.31	0.88–1.94	0.17	-	-	-	1.10	0.78–1.56	0.56	1.10	0.78–1.56	0.56
PET/CT finding	3.35	1.95–5.75	<0.001	3.08	1.79–5.31	<0.001	4.03	2.09–7.77	<0.01	3.92	2.03–7.55	<0.01	3.52	2.05–6.06	<0.01	3.52	2.05–6.06	<0.01

**Table 5:** Univariate and multivariate analysis in all patients (n = 286) assessing predictors of overall survival (OS), cancer specific survival (CCS) and progression free survival (PFS). Model with the most common clinical pathological variables.

	OS						CCS						PFS					
	Univariate			Multivariate			Univariate			Multivariate			Univariate			Multivariate		
	HR	CI95%	p	HR	CI95%	p	HR	CI95%	p	HR	CI95%	p	HR	CI95%	p	HR	CI95%	p
Age	1.02	1.00–1.03	0.018	1.01	0.99–1.03	0.24	1.01	0.99–1.03	0.19	1.01	0.99–1.03		1.01	0.99–1.03	0.05	1.01	0.99–1.03	0.09
Gender (male vs. female)	1.13	0.76–1.68	0.521	0.83	0.55–1.23	0.1.23	1.01	0.64–1.60	0.93	1.05	0.65–1.71	0.83	1.32	0.90–1.92	0.14	0.92	0.60–1.41	0.73
≤pT2 vs. >pT2	1.64	1.14–2.34	<0.01	1.27	0.88–1.84	0.19	1.77	1.19–2.64	<0.01	1.49	0.99–2.26	0.054	1.48	1.04–2.11	0.023	1.41	0.97–2.05	0.068
LN mets (+ vs. −)	1.90	1.30–2.78	<0.01	1.50	1.02–2.19	0.04	1.83	1.20–2.79	<0.01	1.4	0.92–2.21	0.11	1.83	1.26–2.64	<0.01	1.51	1.01–2.25	0.041
PET/CT + vs − findings)	3.35	1.95–5.75	<0.001	3.40	0.2–0.6	<0.01	4.03	2.09–7.77	<0.01	0.3	0.16–0.61	<0.01	3.52	2.05–6.06	<0.01	0.37	0.20–0.65	<0.01

**Table 6:** Univariate and multivariate analysis in patients with only Bladder Cancer (n = 212) assessing predictors of overall survival (OS), cancer specific survival (CCS) and progression free survival (PFS). Only variables set at a p value less than 0.05 in the univariable analysis were included in the model.

	OS						CCS						PFS					
	Univariate			Multivariate			Univariate			Multivariate			Univariate			Multivariate		
	HR	CI95%	p	HR	CI95%	p	HR	CI95%	p	HR	CI95%	p	HR	CI95%	p	HR	CI95%	p
Age	1.01	0.99–1.03	0.12	-	-	-	1.01	0.99–1.03	0.33	-	-	-	1.01	0.99–1.02	0.23	-	-	-
Gender	1.49	0.93–2.38	0.09	-	-	-	1.28	0.73–2.22	0.37	-	-	-	1.45	0.91–2.32	0.11	-	-	-
Neoadjuvant therapy	1.80	1.04–3.14	0.03	1.56	0.88–2.78	0.12	1.82	0.98–3.38	0.06	-	-	-	1.34	0.76–2.36	0.30	-	-	-
Adjuvant/salvage therapies	1.15	0.77–1.72	0.48	-	-	-	1.10	0.70–1.74	0.65	-	-	-	0.99	0.67–1.48	0.98	-	-	-
≤pT2 vs. >pT2	2.16	1.38–3.35	0.01	2.06	1.31-3.22	0.02	2.11	1.28–3.84	0.01	1.91	1.16–3.16	0.01	2.18	1.41–3.37	<0.01	1.88	1.18–2.97	0.01
LN mets	1.34	0.94–1.91	0.10	-	-	-	1.39	0.93–2.08	0.10	-	-	-	1.45	1.01–2.06	0.03	1.31	0.86–1.99	0.20
PET/CT finding	5.16	2.25–11.8	<0.01	4.30	1.86–9.93	<0.01	8.28	2.60–26.3	<0.01	7.27	2.27–23.23	0.01	3.46	1.73–6.89	<0.01	3.06	1.52–6.14	0.02

**Table 7:** Univariate and multivariate analysis in all patients with upper tract urothelial carcinoma (n = 74) assessing predictors of overall survival (OS), cancer specific survival (CCS) and progression free survival (PFS)**.** Only variables set at a p value less than 0.05 in the univariable analysis were included in the model.

	OS						CCS						PFS		
	Univariate			Multivariate			Univariate			Multivariate			Univariate		
	HR	CI95%	p	HR	CI95%	p	HR	IC95%	p	HR	CI95%	p	HR	CI95%	p
Age	1.04	1.00–1.08	0.04	1.02	0.98–1.07	0.18	1.01	0.975–1.06	0.43	-	-	-	1.03	0.99–1.07	0.06
Gender	0.62	0.29–1.32	0.22	-	-	-	0.59	0.25–1.37	0.22	-	-	-	1.25	0.63–2.47	0.51
Neoadjuvant therapy	1.39	0.57–3.39	0.45	-	-	-	1.75	0.70–4.36	0.22	-	-	-	1.20	0.50–2.92	0.67
Adjuvant/salvage therapies	2.05	0.97–4.35	0.05	-	-	-	2.72	1.23–6.00	0.01	2.35	1.05–5.24	0.03	1.59	0.75–3.35	0.22
≤PT2 vs. >PT2	1.25	0.54–2.89	0.59	-	-	-	1.26	0.52–3.05	0.59	-	-	-	0.89	0.41–1.93	0.77
LN mets	1.25	0.87–1.79	0.21	-	-	-	1.27	0.85–1.89	0.23	-	-	-	1.31	0.92–1.86	0.13
PET/CT finding	2.31	1.06–5.02	0.03	1.92	0.84–4.35	0.12	2.44	1.02–5.83	0.04	2.12	0.87–5.17	0.09	3.88	1.57–9.58	0.03

**Table 8:** Univariate and multivariate analysis in patients with only Bladder Cancer (n = 212) assessing predictors of overall survival (OS), cancer specific survival (CCS) and progression free survival (PFS). Model with the most common clinical pathological variables.

	OS						CCS						PFS					
	Univariate			Multivariate			Univariate			Multivariate			Univariate			Multivariate		
	HR	CI95%	p	HR	CI95%	p	HR	CI95%	p	HR	CI95%	p	HR	IC95%	p	HR	CI95%	p
Age	1.01	0.99–1.03	0.12	1.01	0.99–1.03	0.26	1.01	0.99–1.03	0.33	1.00	0.98–1.03	0.56	1.01	0.99–1.02	0.23	1.00	0.98–1.02	0.47
Gender (male vs. female)	1.49	0.93–2.38	0.09	1.30	0.81–2.12	0.27	1.28	0.73–2.22	0.37	1.1	0.62–1.95	0.74	1.45	0.91–2.32	0.11	1.25	0.77–2.04	0.36
≤pT2 vs. >pT2	2.16	1.38–3.35	0.01	1.83	1.14–2.94	0.012	2.11	1.28–3.84	0.01	1.73	1.01–2.94	0.044	2.18	1.41–3.37	<0.01	2.022	1.27–3.22	<0.01
LN mets (positive vs. negative)	1.34	0.94–1.91	0.10	1.21	0.79–1.85	0.39	1.39	0.93–2.08	0.10	1.28	0.79–2.08	0.31	1.45	1.01–2.06	0.03	1.00	6.66–1.55	0.97
PET/CT + vs. − findings)	5.16	2.25–11.8	<0.01	4.29	1.85–9.82	0.01	8.28	2.60–26.3	<0.01	6.96	2.17–22.26	<0.01	3.46	1.73–6.89	<0.01	3.04	1.52–6.08	<0.01

**Table 9:** Univariate and multivariate analysis in all patients with upper tract urothelial carcinoma (n = 74) assessing predictors of overall survival (OS), cancer specific survival (CCS) and progression free survival (PFS)**.** Model with the most common clinical pathological variables.

	OS						CCS						PFS					
	Univariate			Multivariate			Univariate			Multivariate			Univariate			Multivariate		
	HR	CI95%	p	HR	CI95%	p	HR	CI95%	p	HR	CI95%	p	HR	CI95%	p	HR	CI95%	p
Age	1.04	1.00–1.08	0.04	1.05	0.99–1.10	0.08	1.01	0.975–1.06	0.43	1.3	0.98–1.09	0.21	1.03	0.99–1.07	0.06	1.04	0.99–1.08	0.06
Gender (male vs. female)	0.62	0.29–1.32	0.22	0.75	0.28–2.02	0.57	0.59	0.25–1.37	0.22	0.69	0.24–1.99	0.49	1.25	0.63–2.47	0.51	1.88	0.79–4.52	0.15
≤PT2 vs. >PT2	1.25	0.54–2.89	0.59	0.93	0.39–2.24	0.88	1.26	0.52–3.05	0.59	0.98	0.39–2.44	0.96	0.89	0.41–1.93	0.77	0.58	0.29–1.36	0.21
LN mets ( +vs − )	1.25	0.87–1.79	0.21	2.70	1.01–7.18	0.046	1.27	0.85–1.89	0.23	2.83	1.05–7.64	0.039	1.31	0.92–1.86	0.13	3.41	1.35–8.58	<0.01
PET/CT +vs − findings)	2.31	1.06–5.02	0.03	1.28	0.49–3.30	0.60	2.44	1.02–5.83	0.04	1.43	0.52–3.90	0.48	3.88	1.57–9.58	0.03	2.9	1.09–7.74	0.03

## Supplementary Materials

The following are available online at https://www.mdpi.com/2072-6694/11/5/700/s1, Table S1a. Characteristics of patients with only bladder cancer (212/286); Table S1b. Characteristics of patients with only UTUC (38/286); Table S1c. Characteristics of patients with concomitant bladder cancer and UTUC (36/286); Figure S1. ROC curve assessing the accuracy of the multivariabile COX model all patients with and without PET/CT (model with only variables set at a p value less than 0.05 an univariate analysis); Figure S2. ROC curve assessing the accuracy of the multivariabile COX model all patients with and without PET/CT (model with the most common clinical-pathological variables); Figure S3. ROC curve assessing the accuracy of the multivariabile COX model for bladder cancer with and without PET/CT (model with only variables set at a p value less than 0.05 an univariate analysis); Figure S4. ROC curve assessing the accuracy of the multivariabile COX model for bladder cancer with and without PET/CT (model with the most common clinical-pathological variables); Figure S5. ROC curve assessing the accuracy of the multivariabile COX model for upper tract tumors with and without PET/CT (model with the most common clinical-pathological variables); Figure S6. PET/CT is able to stratify recurrence free survival for those patients with a FP and FN at conventional imaging; Figure S7. PET/CT is able to stratify overall survival for those patients with a FP and FN conventional imaging
