# Supplementary material for: 18F-FDG PET/CT and Urothelial Carcinoma: Impact on Management and Prognosis—A Multicenter Retrospective Study
Source: Cancers (Basel). 2019 May 20;11(5):700. doi: 10.3390/cancers11050700 (PMC6562413; doi:10.3390/cancers11050700)
Supplement: Supplementary file 1 [file cancers-11-00700-s001.pdf]

Supplementary materials

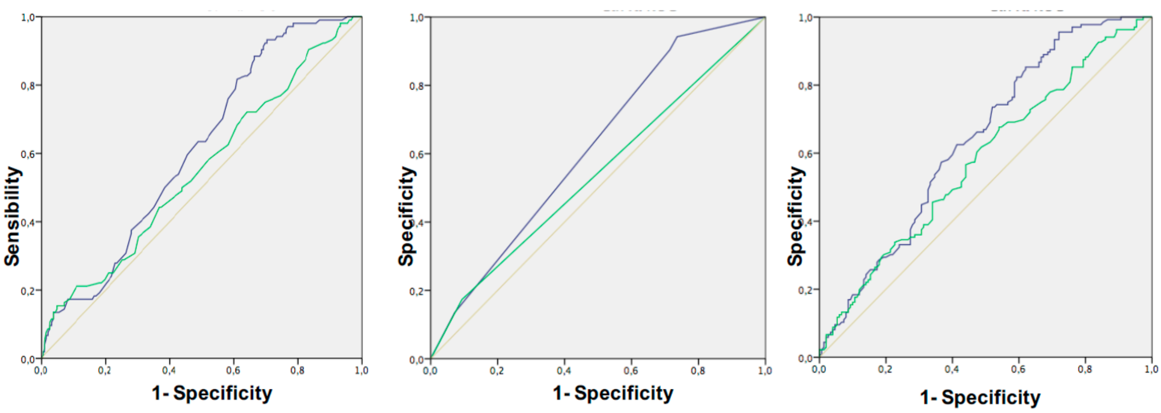

	OS	CSS	PFS
**Model with PET/CT**	0.61	0.61	0.64
**Model without PET/CT**	0.55	0.54	0.58

Figure S1. ROC curve assessing the accuracy of the multivariabile COX model all patients with and without PET/CT (model with only variables set at a p value less than 0.05 an univariate analysis).

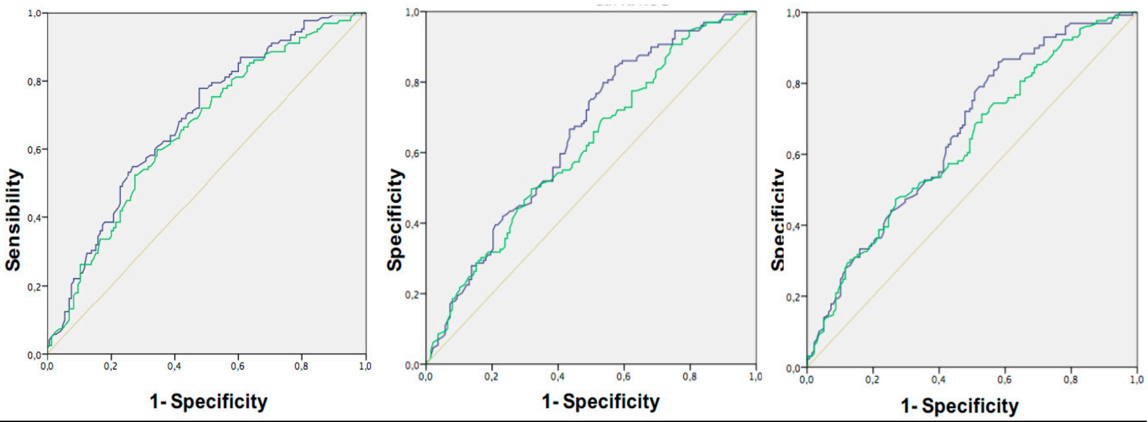

	OS	CSS	PFS
**Model with age, sex, pT, pN, PET/CT (blue line)**	0.68	0.66	0.66
**Model with pT, pN, age,sex (green linee)**	0.65	0.62	0.63

Figure S2. ROC curve assessing the accuracy of the multivariabile COX model all patients with and without PET/CT (model with the most common clinical-pathological variables).

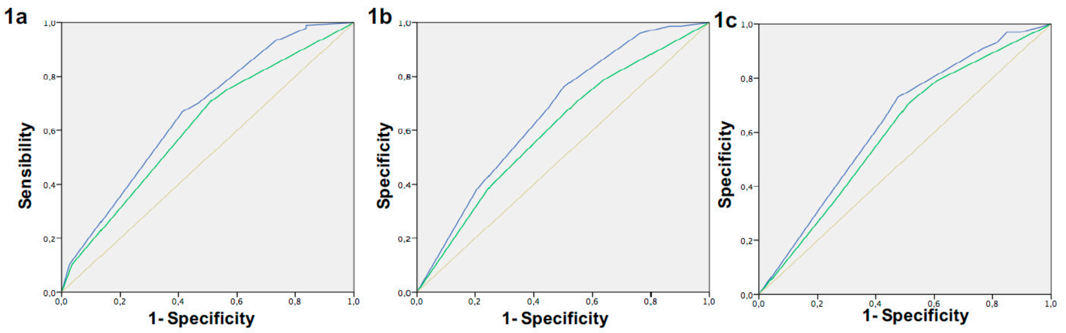

	OS	CSS	PFS
Model with PET/CT (blue line)	0.66	0.67	0.64
Model without PET/CT (green linee)	0.61	0.60	0.60

Figure S3. ROC curve assessing the accuracy of the multivariabile COX model for bladder cancer with and without PET/CT (model with only variables set at a p value less than 0.05 an univariate analysis).

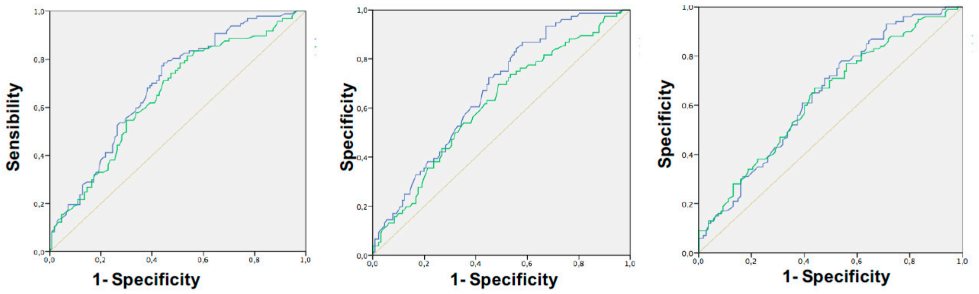

	OS	CSS	PFS
Model with age, sex, pT, pN, PET/CT (blue line)	0.69	0.67	0.64
Model with pT, pN, age,sex (green linee)	0.65	0.61	0.63

Figure S4. ROC curve assessing the accuracy of the multivariabile COX model for bladder cancer with and without PET/CT (model with the most common clinical-pathological variables).

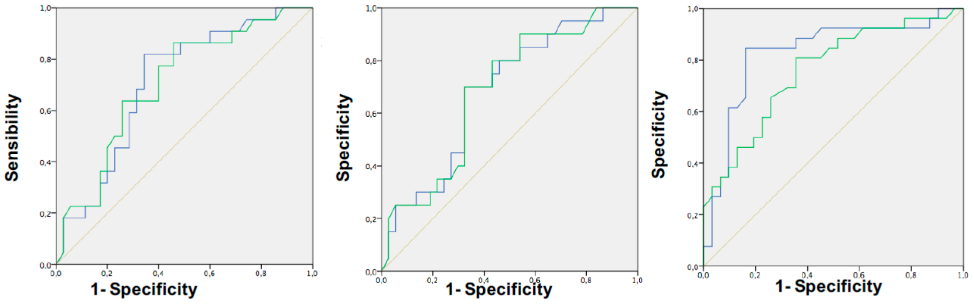

	OS	CSS	PFS
Model with age, sex, pT, pN and PET/CT	0.71	0.68	0.83
Model with pT, pN, age and sex	0.70	0.68	0.76

Figure S5. ROC curve assessing the accuracy of the multivariabile COX model for upper tract tumors with and without PET/CT (model with the most common clinical-pathological variables).

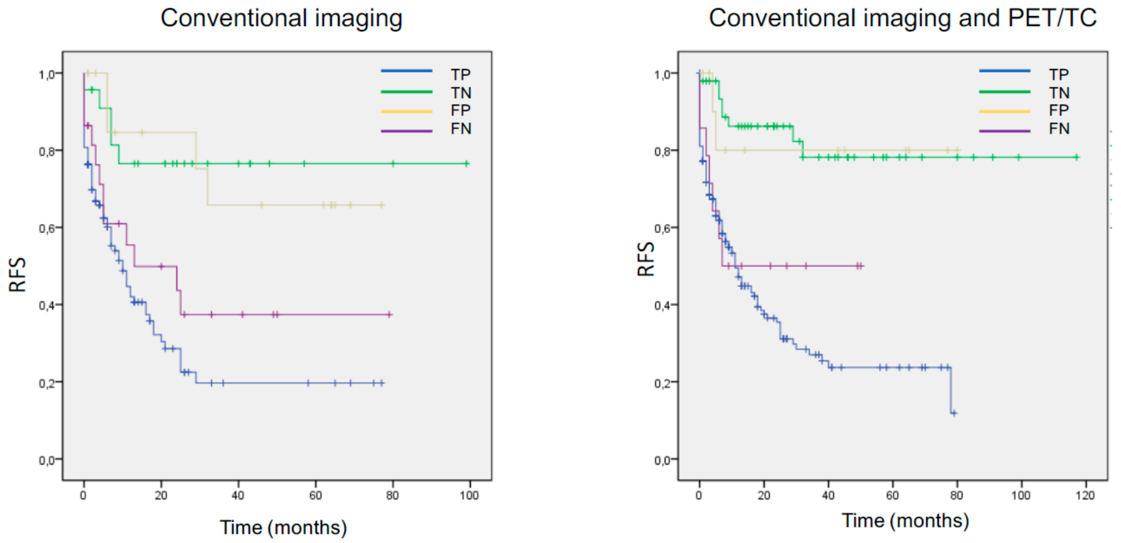

Figure S6. PET/CT is able to stratify recurrence free survival for those patients with a FP and FN at conventional imaging.

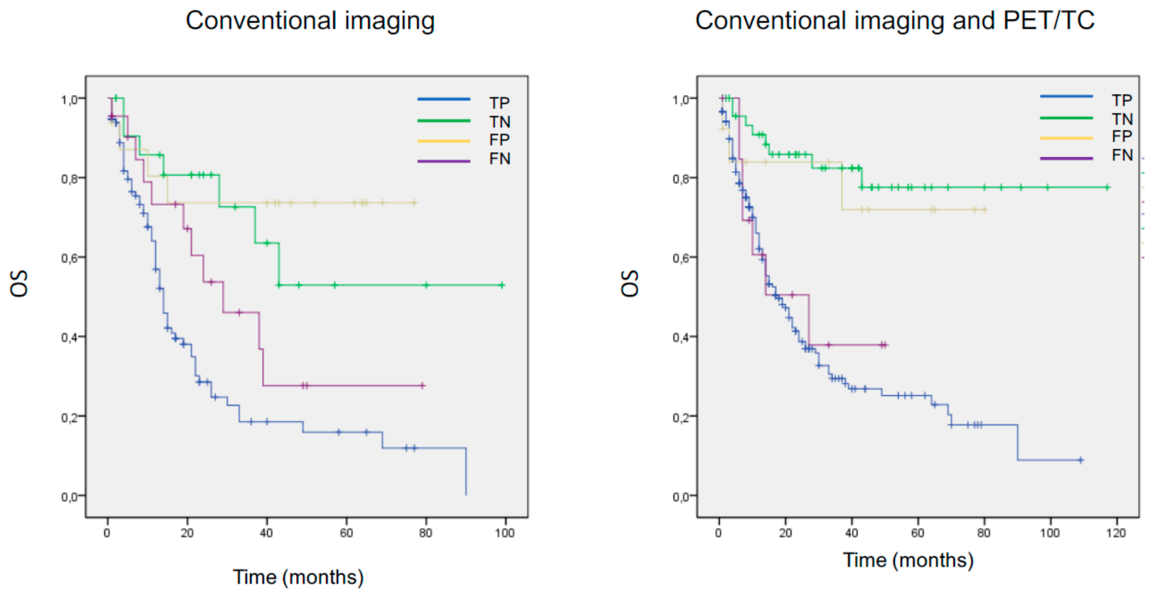

Figure S7. PET/CT is able to stratify overall survival for those patients with a FP and FN conventional imaging.

Table S1a. Characteristics of patients with only bladder cancer (212/286).

**Variables**	**No.**
pT of bladder cancer, n (%)	
pT ≤ 2	82/212 (40.0)
pT 3–4	126/212 (59.4)
NA	4/212 (1.8)
Tumor grade	
Low Grade	15/212 (7.0)
High grade	197/212 (91.0)
N/A	4/212 (1.8)
pN of bladder cancer, n (%)	
pNx	3/212 (1.4)
pN0	125/212 (59)
pN1	29/212 (13.7)
pN2	43/212 (20.3)
pN3	9/212 (4.2)
NA	3/212 (1.4)
Mean of removed Lymph nodes of bladder cancer (IQR)	20 (12–27)
Mean of metastatic lymph nodes of bladder cancer (IQR)	2 (0–1.75)
Neoadjuvant treatments, n (%)	
No	187/212 (88.2)
Yes	25/212 (11.8)
Adjuvant treatments, n (%)	
No	119/212 (56.1)
Yes	93/212 (43.9)
Type of adjuvant treatments, n (%)	
No	119/212 (56.1)
Chemotherapy	75/212 (35.4)
Radiotherapy	17/212 (8)
Combination of chemotherapy and radiotherapy	1/212 (0.5)

Table S1b. Characteristics of patients with only UTUC (38/286).

Variables	No.
pT of UUTC (*), n (%)	
pT ≤ 2	13/38 (34.2)
pT 3–4	16/38 (42.1)
NA	9/38 (23.7)
Tumor grade	
Low Grade	1/38 (2.6)
High grade	27/38 (96.4)
pN of UUTC (*), n (%)	
pNx	19/38 (50.0)
pN0	14/38 (36.8)
pN1	2/38 (5.2)
pN2	3/38 (7.9)
Mean of removed Lymph nodes of UUTC (*) (IQR)	4 (0–8.5)
Mean of metastatic lymph nodes of UUTC (*) (IQR)	1(1–2)
UUTC (*) location, n (%)	
pelvis	10/38 (26.3)
ureter	23/38 (60.5)
multifocality	5/38 (13.1)
UUTC (*) treatments, n (%)	
Nephroureterectomy	35/38 (92.1)
Other treatments	3/38 (7.9)
Neoadjuvant treatments, n (%)	
No	33/38 (86.8)
Yes	5/38 (13.1)
Adjuvant treatments, n (%)	
No	30/38 (78.9)
Yes	8/38 (21.1)
Type of adjuvant treatments, n (%)	
No	30/38 (72.2)
Chemotherapy	7/38 (18.4)
Radiotherapy	0
Combination of chemotherapy and radiotherapy	1/38 (2.6)

* Upper urinary tract carcinoma.

Table S1c. Characteristics of patients with concomitant bladder cancer and UTUC (36/286).

Variables	No.
Radical cystectomy, n (%)	
Open cystectomy	35/36 (97.2)
Robotic cystectomy	1/36 (2.8)
pT of bladder cancer, n (%)	
pT ≤ 2	21/36 (58.3)
pT 3–4	11/36 (30.5)
NA	4/36 (11.1)
Tumor grade	
Low Grade	2/36 (5.5)
High grade	30/36 (83.3)
N/A	4/36 (11.1)
pN of bladder cancer, n (%)	
pNx	1/36 (2.8)
pN0	22/36 (61.1)
pN1	4/36 (11.1)
pN2	4/36 (11.1)
pN3	1/36 (2.8)
NA	4/36 (11.1)
Mean of removed Lymph nodes of bladder cancer (IQR)	20 (12–27)
Mean of metastatic lymph nodes of bladder cancer (IQR)	1 (0–1)
pT of UUTC (*), n (%)	
pT ≤ 2	17/36 (47.2)
pT 3–4	12/36 (33.3)
NA	7/36 (19.4)
pN of UUTC (*), n (%)	
pNx	31/36(86.1)
pN0	4/36(11.1)
pN1	1/36 (2.8)
Mean of removed Lymph nodes of UUTC (*) (IQR)	10 (0–16)
Mean of metastatic lymph nodes of UUTC (*) (IQR)	1 (1–2)
UUTC (*) location, n (%)	
pelvis	20/36 (55.5)
ureter	9/36 (25)
multifocality	7/36 (19.4)
UUTC (*) treatments, n (%)	
Nephroureterectomy	31/36 (86.1)
Other treatments	5/36 (13.9)
Neoadjuvant treatments, n (%)	
No	31/36 (86.1)
Yes	5/36 (13.9)
Adjuvant treatments, n (%)	
No	26/36 (72.2)
Yes	10/36 (27.8)
Type of adjuvant treatments, n (%)	
No	26/36 (72.2)
Chemotherapy	7/36 (19.4)
Radiotherapy	2/36 (5.6)
Combination of chemotherapy and radiotherapy	1/36 (2.8)

* Upper urinary tract carcinoma.
